# Post-treatment of rat aflatoxicosis by camel milk and silymarin

**DOI:** 10.3389/fphar.2025.1513105

**Published:** 2025-02-11

**Authors:** Nahla H. Hassaneen, Shabaan A. Hemeda, Abeer F. El Nahas, Ghadeer M. Albadrani, Muath Q. Al-Ghadi, Zuhair M. Mohammedsaleh, Sabreen E. Fadl, Eman M. El-Diasty, Hader I. Sakr

**Affiliations:** ^1^ Department of Animal Husbandry and Animal Wealth Development, Faculty of Veterinary Medicine, Matrouh University, Matrouh, Egypt; ^2^ Department of Animal Husbandry and Animal Wealth Development, Faculty of Veterinary Medicine, Alexandria University, Alexandria, Egypt; ^3^ Department of Biology, College of Science, Princess Nourah bint Abdulrahman University, Riyadh, Saudi Arabia; ^4^ Department of Zoology, College of Science, King Saud University, Riyadh, Saudi Arabia; ^5^ Department of Medical Laboratory Technology, Faculty of Applied Medical Sciences, University of Tabuk, Tabuk, Saudi Arabia; ^6^ Biochemistry Department, Faculty of Veterinary Medicine, Matrouh University, Matrouh, Egypt; ^7^ Mycology Department, Animal Health Research Institute Dokki, Giza (ARC), Giza, Egypt; ^8^ Department of Medical Physiology, Faculty of Medicine, Cairo University, Giza, Egypt; ^9^ Department of Medical Physiology, General Medicine Practice Program, Batterjee Medical College, Jedda, Saudi Arabia

**Keywords:** aflatoxin B1, post-treatment, camel milk, silymarin, serum biochemistry, gene expression

## Abstract

**Background:**

Aflatoxins are highly potent mycotoxins that can seriously harm the health of humans and a variety of animal species. On the other hand, camel milk and silymarin offer a variety of positive effects for many animal species. In addition, camel milk and silymarin reduce the impact of AFB1 on the hematology, serum biochemical markers, histopathology of the liver and testes, and expression of the inflammatory, antioxidant, and male reproductive genes.

**Methods:**

40 rats were used to evaluate the beneficial effect of silymarin and camel milk against aflatoxin B1 (AFB1) toxicity in rats. The classified treatments were the control negative (no treatment) and the control positive (supplied with 1.4 mg aflatoxin/kg diet) for 28 days. Camel milk group (supplied with 1.4 mg aflatoxin/kg diet) for 28 days and camel milk (1 milliliter of camel milk per kilogram of body weight) orally, from day 29 to day 43). Silymarin (supplied with 1.4 mg aflatoxin/kg diet) for 28 days and silymarin (20 mg silymarin/kg b.wt), orally, from day 29 to day 43). The evaluation was done through measuring leukocyte count, liver function tests, carcinoembryonic antigen (CEA), alpha-fetoprotein (AFP), ferritin, and testosterone. Moreover, the histopathology of the liver and testes was done along with expression levels of specific genes in the liver and testes.

**Results:**

The outcomes showed that the post-treatment with silymarin and camel milk improved biochemical markers in serum and ability to reproduce.

**Conclusion:**

In conclusion, post-treatment with camel milk and silymarin could mitigate the negative effect of AFB1 on rats.

## Introduction

*Aspergillus flavus* and *A. parasiticus* are two species of *Aspergillus* that generate mycotoxin as their secondary metabolite (Ali et al., 2022; Saleemi et al., 2023). Aflatoxin B1 (AFB1), the most dangerous aflatoxin, is a key contributor to hepatocellular cancer (Rotimi et al., 2021). *A. flavus*-produced mycotoxins are frequently found on grains that have been harvested and stored damp. It can also be detected on grains that have been grown under stressful conditions, such as high moisture and temperature and on cereals like wheat, corn, beans, and rice (Kudayer et al., 2019). AFB1 can result in oxidative stress, growth inhibition, and liver damage (Schrenk et al., 2020). It has significant DNA mutagenicity and carcinogenicity, and the International Agency for Research on Cancer (IARC) has categorized it as a group-1 carcinogen (Kilic et al., 2022). Moreover, AFB1 has been widely reported to be hepatotoxic during mammalian growth and development (Wu et al., 2022). It is well recognized that aflatoxins are strong mutagenic, hepatotoxic, hepatocarcinogenic, nephrotoxic, teratogenic, genotoxic, and immunosuppressive and that they also impede several metabolic processes, thus harming the heart, liver, and kidneys (Yilmaz et al., 2018; Altyar et al., 2023; 2024). These poisons have been implicated as the cause of some human deaths and excessive livestock mortality (Ijaz et al., 2022). Excessively vacuolated cells and spermatogenesis suppression were observed in the testicles of aflatoxicated rats (Kudayer et al., 2019).

Milk is crucial to maintaining health and preventing disease (Meisel, 2005; Yahya et al., 2018). Milk from camels and cows is a vital source of crucial nutrients (Saeed et al., 2022). Many diabetic people require camel milk for medical help, and it is also employed as an anti-microbial hepatoprotective medication due to its unique properties (Althnaian et al., 2013). In addition to minerals and vitamins, camel milk also contains several useful biological components (Albarrak, 2023). The treatment of various immunological abnormalities and metabolic dysfunctions, particularly those linked to some kinds of diabetes, has shown positive medical results (Agrawal et al., 2002; 2007; Mohamad et al., 2009). Additionally, camel milk is used to treat several chronic illnesses (Alorainy et al., 2023). Numerous studies claim that camel milk protects against the harmful effects of toxic substances like cadmium chloride, aluminum chloride, paracetamol, carbon tetrachloride, and cisplatin (Al-Asmari et al., 2017; Al-Fartosi et al., 2011; Afifi, 2010; Al-Hashem et al., 2009; Dallak, 2009; Al-Hashem, 2009).

Natural remedies made from plants are being used more frequently across the world because they are seen to be potentially safe and effective alternatives to conventional therapies for liver problems (Wang et al., 2018). *Silybum marianum* (Asteraceae) has a long history of use as a treatment for a variety of hepatic illnesses, including cirrhosis and hepatitis, as well as preventing liver damage arising from toxins and chemicals in the environment (Abenavoli et al., 2010; Pascual et al., 1993). Silymarin, a hepatoprotective substance that has demonstrated protective properties against inflammation, oxidation, and apoptosis, has been used clinically for centuries, either by itself or as a main component of a variety of medicinal formulations (Arafa, 2009; Shuppan et al., 1999). Silymarin’s stabilizing impact on cytoplasmic membranes is linked to its anti-hepatotoxic action (Wang et al., 2018).

Previous studies (Hassaneen et al., 2023; Hassaneen et al., 2024) have discussed the therapeutic effect of camel milk and silymarin on the harmful effect of aflatoxin in rats from the onset of poisoning. Therefore, this experiment was conducted to evaluate post-treatment with camel milk and silymarin for 14 days on a male rat model exposed to aflatoxin toxicity for 28 days in the diet.

## Materials and methods

### Experimental animals and design

**FIGURE 1:**
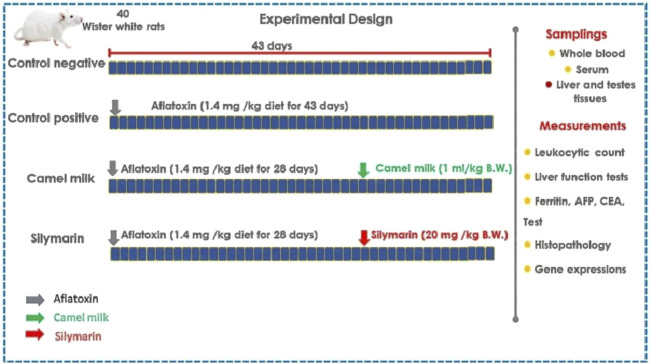
Experimental design.

The Faculty of Veterinary Medicine, Alexandria University, Egypt, gave its ethical approval to this work. We acquired 40 mature (4 weeks old) male (Wister white) rats (84.7 g; Hassaneen et al. (2024)) from the Abdo farm for laboratory animals located in Alexandria, Egypt, for this study (Figure 1). We were granted permission to use the rats after formally applying to the Faculty of Veterinary Medicine, Matrouh University. During the acclimatization (15 days) and experimental periods (43 days), water was available at all times. The aflatoxicated diet was prepared using autoclaved crushed yellow corn, where 10 mL of the toxigenic strain’s spore suspension (10^6^ spores/mL) was added. After that, the treated corn was incubated for 21 days at 28–30°C to ferment it. After 21 days, the corn was incubated for 3–4 days at 50°C to kill the fungus. The crushed corn was then ground into a powder using the grinder. Next, AFB1 was calculated using a representative sample of 25 g powdered corn (AOAC, 1980). To obtain the required dosage of 1.4 mg of aflatoxin/kg of feed, the tainted corn was then mixed with commercially crushed corn previously measured for the presence of mycotoxins. All rats were given free access to this prepared feed. They were classified into four groups of ten rats each. The classified groups were the control negative (no medical attention) and control positive (administered aflatoxin 1.4 mg/kg feed for 28 days, as per El-Nekeety et al. (2014)). The camel milk group was administered aflatoxin 1.4 mg/kg feed for 28 days and 1 mL of camel milk per kilogram of body weight administered orally as per Al-Hashem et al. (2009) from days 29 to 43. The silymarin group was administered aflatoxin 1.4 mg/kg feed for 28 days and oral silymarin (20 mg/kg b.wt) suspension, as per Rastogi et al. (2001), from days 29 to 43.

After 43 days (end of the trial), whole blood with EDTA and serum samples were collected from the medial canthus of the eye of all groups after isoflurane anesthesia. The whole blood was used to count leukocytes and deferential. Meanwhile, serum was stored at −20°C to measure aminotransferases (AST and ALT) and the proteins’ calorimetric and carcinoembryonic antigen (CEA), alpha-fetoprotein (AFP), ferritin, and testosterone by a Microplate Immunosymmetric assay. Liver and testis tissue samples (n = 5) were collected after cervical dislocation for histopathological examination in formalin. The sections of the liver and testes were collected and immediately fixed in 10% buffered formalin and processed for histopathological evaluation using routine paraffin sections. Sections 5 μm thick were cut and stained with hematoxylin and eosin (H&E) as per Bancroft and Gamble (2008). To pathologically score the liver and testes, eight X200 power fields with a total area of roughly 11–12 mm^2^ were examined. On a three-point rating system, lesions of the liver and testes were evaluated by adding the following factors: necrosis, degenerative and inflammatory alterations, and the diffusion of the lesions. In accordance with normal tissue, mild, moderate, severe (multi focused), and more serious lesions (diffuse), the scores for each parameter ranged from 0 to 4. Moreover, the tissue samples from liver and testes were collected for gene expression and stored at −08°C until RNA isolation.

### Gene expression

We employed quantitative real-time PCR (qRT-PCR) to obtain the expression of: *NQO 1*, NAD(P)H quinone dehydrogenase 1; *TNFα*, tumor necrosis factor α; *APE1*, apurinic/apyrimidinic endodeoxyribonuclease 1; *OGG1*, 8-oxoguanine DNA glycosylase; *LHR,* luteinizing hormone receptor; *StAR*, steroidogenic acute regulatory protein. The liver and testicular samples were obtained, frozen in liquid nitrogen, and kept at −80°C to extract RNA. Using an RNA Purification Kit from Thermo Scientific (USA), RNA was isolated from the frozen samples in accordance with the manufacturer’s instructions. To produce cDNA from a fixed concentration of RNA, Thermo Scientific USA’s Intron-Power cDNA synthesis kit was utilized. Using the β-actin housekeeping gene as a normalization, the negative control group was the calibrator. Specific primers (Table 1) were utilized to amplify *NQO 1, TNFα, APE1, LHR*, and *StAR* in rats to assay the qRT-PCR. The 2^−ΔΔCt^ approach was utilized to analyze the data acquired (Livak and Schmittgen, 2001).

**TABLE 1:** Primers used in this study.

Genes	Primer’s sequence (5′–3′)	Acc. Number
*NQO 1*	F: ACC​TCT​CTG​TGG​TTT​AGG​GC	NM_017000.3 (Xie et al., 2018)
	R: GGA​CCT​GGG​TGT​GCT​ATG​TA	
*TNFα*	F: CCA​CGT​CGT​AGC​AAA​CCA​CCA​AG	NM_012675.3 (Gong et al., 2018)
	R: CAG​GTA​CAT​GGG​CTC​ATA​CC	
*APE1*	F: GCT​CAG​AGA​ACA​AAC​TCC​CG	XM_017599805.2 (Luceri et al., 2018)
	R: TTG​TTT​CCT​TTG​GGG​TTA​CG	
*OGG1*	F: CCT​GGC​TGG​TCC​AGA​AGT​AG	XM_039108420.1 (Luceri et al., 2018)
	R: TTT​CCC​AGT​TCT​TTG​TTG​GC	
*LHR*	F: CCA​GAA​CAC​CAA​AAA​CCT​GCT	NM_012978 (Elblehi et al., 2019)
	R: ATC​TGG​AAG​GGT​TCG​GAT​GC	
*StAR*	F: GCCTGCAATTTGGTGGA	NM_031558 (Shi et al., 2007)
	R: GGG​CAT​ACT​CAA​CAA​CCA​G	
*β-actin*	F: CAC​CAT​GTA​CCC​AGG​CAT​TG	NM_031144.3 (Xie et al., 2018)
	R: ACA​GTC​CGC​CTA​GAA​GCA​TT	

*NQO 1*, NAD(P)H quinone dehydrogenase 1; *TNFα*, tumor necrosis factor α; *APE1*, apurinic/apyrimidinic endodeoxyribonuclease 1; *OGG1*, 8-oxoguanine DNA glycosylase, *LHR,* luteinizing hormone receptor; *StAR*, steroidogenic acute regulatory protein.

### Statistical analysis

Data were analyzed by one way analysis of variance (ANOVA), where all results are reported as means ± SEM. Shapiro–Wilk and Levene testing confirmed normal distribution and variance homogeneity. GraphPad Prism version 7.00 for Windows, GraphPad Software, La Jolla, California, United States, www.graphpad.com, was used to perform statistical analysis and create graphs. The significance level was *P* ≤ 0.05.

## Results

### Leukogram

Table 2 illustrates the impact of camel milk and silymarin post-treatment on leukocytic count in rats given diets containing 1.4 mg of aflatoxin B1/kg diet for 43 days. The counts of WBC, lymphocytic, monocyte, and basophil decreased in the control positive group as opposed to the control negative; these decreases were significant (*P* ≤ 0.05). Meanwhile, the neutrophil count significantly (*P* ≤ 0.05) increased in the control positive as opposed to the negative. On the other hand, the eosinophil count increased in the control positive group as opposed to the negative control without significance (*P* ≤ 0.05). Both post-treatments (camel milk and silymarin) increased counts of WBC, lymphocyte, and basophil compared to the positive control group. Meanwhile, the results for eosinophils and monocytes were significantly (*P* ≤ 0.05) increased only in the silymarin group, as opposed to the positive control. On the other hand, the neutrophil count significantly (*P* ≤ 0.05) decreased in both post-treatment groups compared to the positive control.

**TABLE 2:** Effect of treatments on leukocytic count in aflatoxin-exposed rats (n = 10).

Items Groups	WBCs count (×10^3^/µL)	LYM (×10^3^/µL)	NET (×10^3^/µL)	ESI (×10^3^/µL) 2	MON (×10^3^/µL) 1	BAS (×10^3^/µL) 3
Control negative	11.1 ± 0.27^a^	86.40 ± 1.32^a^	9.00 ± 0.44^b^	0.96 ± 0.05^b^	3.40 ± 0.24^a^	0.24 ± 0.02^a^
Control positive	8.08 ± 0.22^c^	69.60 ± 2.03^b^	23.40 ± 0.50^a^	1.16 ± 0.06^b^	2.60 ± 0.24^b^	0.04 ± 0.02^b^
Camel milk	10.10 ± 0.18^b^	87.0 ± 1.78^a^	8.80 ± 0.58^b^	1.16 ± 0.10^b^	3.00 ± 0.32^ab^	0.24 ± 0.02^a^
Silymarin	9.48 ± 0.51^b^	85.80 ± 2.35^a^	9.20 ± 0.49^b^	1.40 ± 0.03^a^	3.40 ± 0.24^a^	0.20 ± 0.03^a^

Values are means ± standard error. Mean values with different subscript letters (a-d) at the same column significantly differ at (*P* ≤ 0.05). WBCs, white blood cells; LYM, lymphocyte; NET, neutrophil; ESI, eosinophil; MON, monocytes; BAS, basophil.

### Liver function

Table 3 illustrates the impact of post-treatment camel milk and silymarin on liver function in rats given diets containing 1.4 mg aflatoxin B1/kg for 43 days. There was a significant (*P* ≤ 0.05) increase in the serum concentration of AST and ALT in the control positive group compared with the control negative. Meanwhile, serum proteins decreased in the control positive groups without significance (*P* ≤ 0.05) as opposed to the negative. The post-treatment outcomes showed that the serum ALT and AST significantly (*P* ≤ 0.05) decreased in the group that received camel milk as opposed to the positive control. Meanwhile, AST only in the silymarin group significantly (*P* ≤ 0.05) decreased as opposed to the positive control. Serum proteins increased in the camel milk and silymarin groups as opposed to the positive control without significance (*P* ≤ 0.05), except that serum albumin significantly (*P* ≤ 0.05) increased in the camel milk group.

**TABLE 3:** Effect of treatments on liver function in aflatoxin-exposed rats (n = 10).

Items Groups	ALT (U/L)	AST (U/L)	T. Protein (g/dL)	Albumin (g/dL)	Globulin (g/dL)
Control negative	77.0 ± 1.4^b^	307.4 ± 2.7^d^	7.2 ± 0.1^a^	4.1 ± 0.1^ab^	3.2 ± 0.2^a^
Control positive	83.0 ± 1.3^a^	410.2 ± 2.6^a^	6.9 ± 0.1^a^	3.6 ± 0.2^b^	2.4 ± 0.2^a^
Camel milk	64.8 ± 1.5^c^	348.8 ± 2.9^c^	7.2 ± 0.4^a^	4.2 ± 0.1^a^	2.8 ± 0.1^a^
Silymarin	79.0 ± 1.4^ab^	388.6 ± 1.7^b^	7.0 ± 0.1^a^	4.1 ± 0.1^ab^	2.9 ± 0.0^a^

Values are means ± standard error. Mean values with different subscript letters (a-d) at the same column significantly differ at (*P* ≤ 0.05).

### Serum biochemical markers

Table 4 illustrates the impact of camel milk and silymarin post-treatment on serum biochemical measurements in rats given diets containing 1.4 mg aflatoxin B1/kg for 43 days. The concentrations of AFP and CEA increased significantly (*P* ≤ 0.05) in the control positive group as opposed to the control negative, while serum ferritin decreased in the control positive group without significance (*P* ≤ 0.05) compared to control negative. On the other hand, the testosterone result decreased significantly (*P* ≤ 0.05) in the control positive group as opposed to the control negative. For post-treatment, the concentrations of AFP and CEA of the post-treatment groups (camel milk and silymarin) significantly (*P* ≤ 0.05) decreased as opposed to the positive control. Moreover, testosterone significantly (*P* ≤ 0.05) increased in the camel milk and silymarin groups compared to the control positive. Meanwhile, serum ferritin significantly (*P* ≤ 0.05) increased in the camel milk group as opposed to the positive control one, while it increased without significance (*P* ≤ 0.05) in the silymarin group.

**TABLE 4:** Effect of treatments on biochemical markers in aflatoxin-exposed rats (n = 10).

Items Groups	Ferritin (mg/dL)	Alpha fetoprotein (ng/mL)	Carcinoembryonic antigen (CEA) ng/mL	TESTO (ng/dL)
Control negative	1.6 ± 0.34^b^	8.7 ± 0.2^b^	0.6 ± 0.0^b^	2.9 ± 0.2^a^
Control positive	1.5 ± 0.1^b^	10.6 ± 0.2^a^	0.9 ± 0.1^a^	1.4 ± 0.1^b^
Camel milk	2.4 ± 0.2^a^	4.9 ± 0.2^c^	0.6 ± 0.00^b^	3.3 ± 0.1^a^
Silymarin	1.8 ± 0.4 ^ab^	4.1 ± 2.2^c^	0.5 ± 0.0^b^	3.0 ± 0.3^a^

Values are means ± standard error. Mean values with different subscript letters (a-d) at the same column significantly differ at (*P* ≤ 0.05).

### Histopathology of liver and testes

**FIGURE 2:**
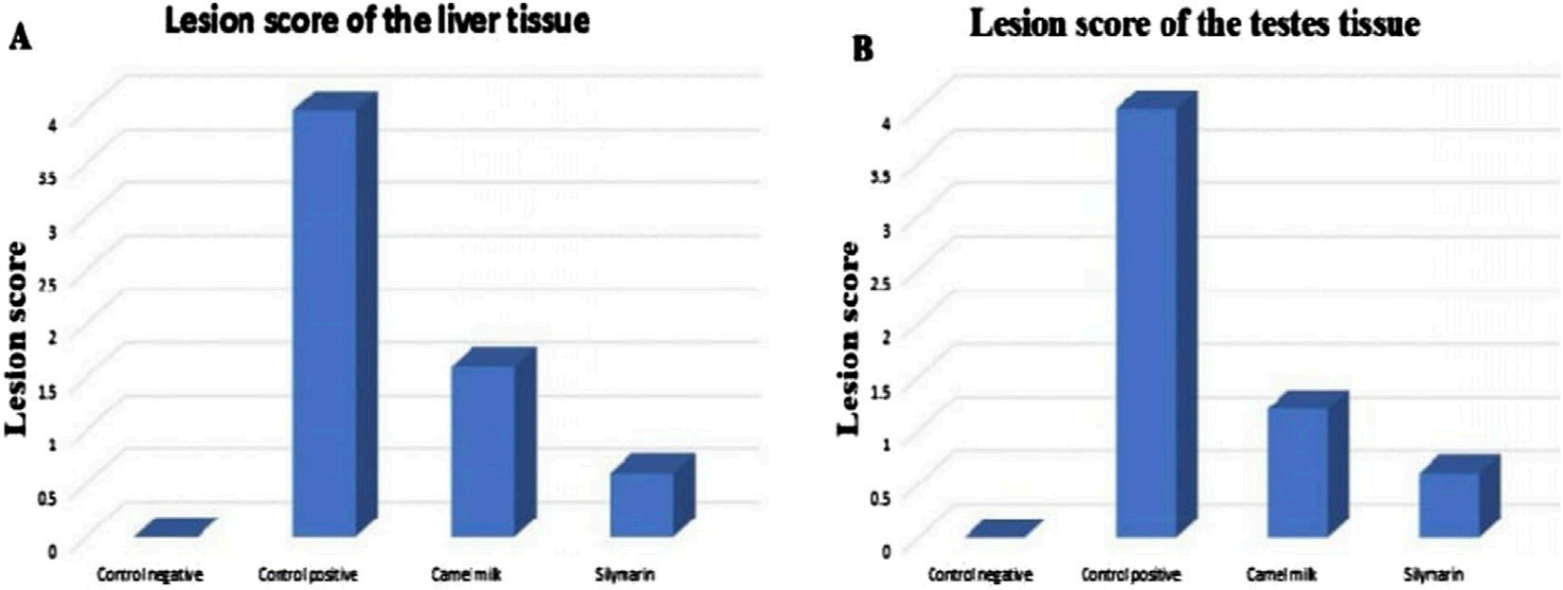
Lesion score of the liver and testis tissues in different groups. **(A)** Lesion score of the liver tissue. **(B)** Lesion score of the testis tissues.

The quantitative assessment of the lesion scores in the liver and testis tissues regarding aflatoxin supplementation and administration of camel milk and silymarin is shown in Figure 2. The rats supplemented with a control diet had normal tissue. Rats supplemented with the aflatoxicated diet showed the most severe damage to their liver and testis tissues. However, the lesion scores significantly decreased in rats administered camel milk and silymarin.

**FIGURE 3:**
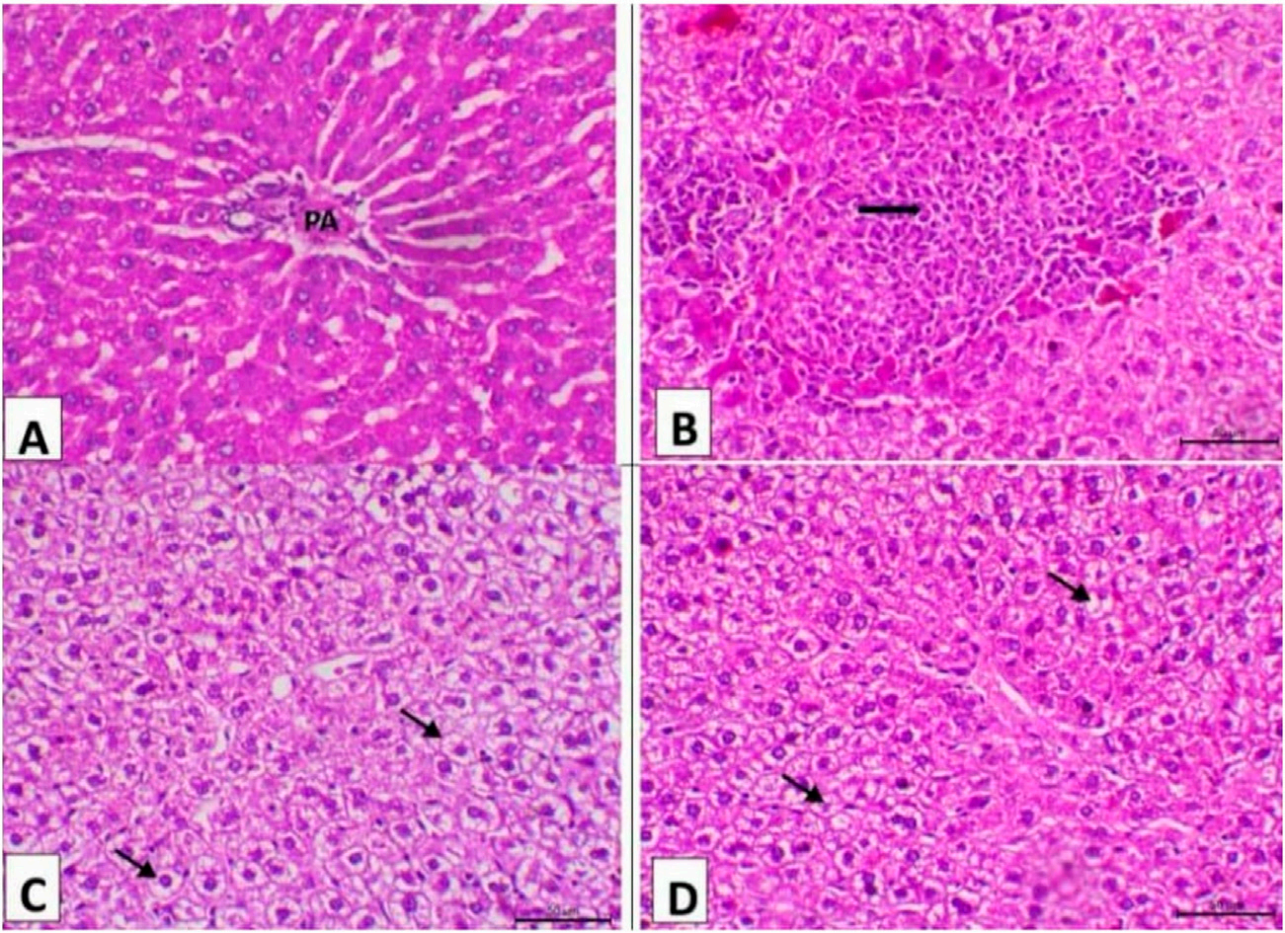
Photomicrograph of the liver (n = 5) of the experimental groups (H&E, X200, Scale bar = 50 μ). **(A)** Liver of the control negative group showing normal hepatic histology featuring normal hepatocytes arranging in cords and normal portal area (PA). **(B)** Liver of the control positive group showing marked fibrosis of the portal area and a high level of mononuclear cell infiltration in the portal area. **(C)** Liver of the camel milk group showing decrease the vacuolar degeneration of the hepatocytes (arrows). **(D)** Liver of the silymarin group showing marked decrease the vacuolar degenerative changes of the hepatocytes (arrows).

**FIGURE 4:**
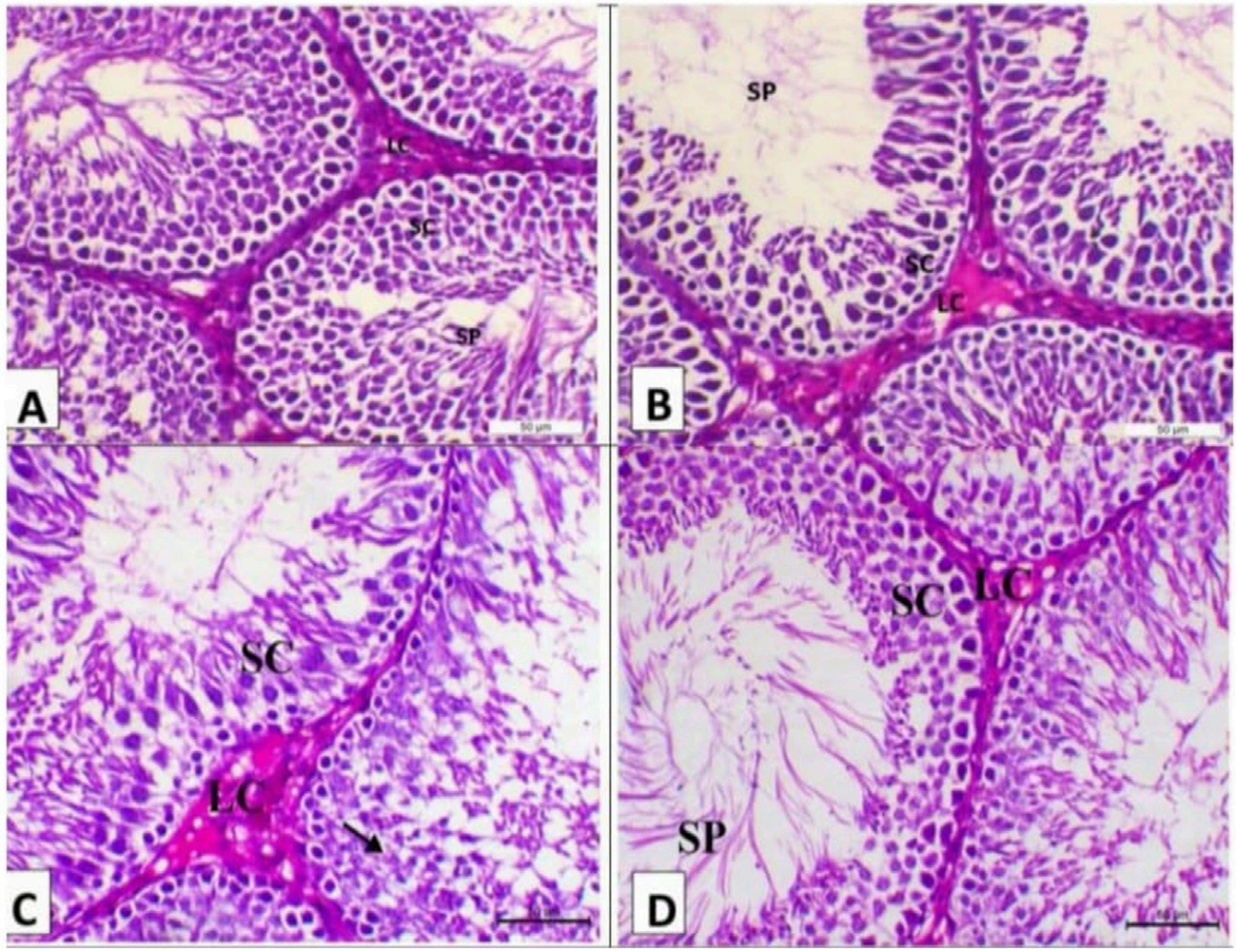
Photomicrograph of the testes (n = 5) of the experimental groups (H&E, X200, Scale bar 50p). **(A)** Testes of the control negative group showing normal testicular histology with the seminiferous tubule lined with multilayer of spermatogenic cells (SC) and impacted with sperm (arrow) with interstitial Leydig cells (LC). **(B)** Testes of the control positive group showing arrest of spermatogenesis in some seminiferous tubules with degenerative changes of spermatogenic cells and interstitial edema. **(C)** Testes of the camel milk group showing decrease in the vacuolar degeneration of the spermatogenic cells (arrows) with decrease in the oedema within the interstitial tissues (SC indicates spermatogenic cells and LC indicates Leydig cells). **(D)** Testis of the silymarin group showing a marked decrease in the degenerative changes and mostly revealing normal testicular histology as the seminiferous tubule lined with a multilayer of spermatogenic cells (SC) and impacted with sperms (SP) with interstitial Leydig cells (LC).

The result of the liver and testicular tissue histopathology is shown in Figures 3 and 4 at 15 days post-treatment. The control group’s liver displayed normal hepatic histology, with a normal portal region and normal hepatocyte arrangement in cords (PA) (Figure 3A). The control positive group demonstrated significant portal area fibrosis and a high level of mononuclear cell infiltration in the portal area (Figure 3B). However, camel and silymarin helped lessen these adverse effects, the liver of the camel milk group showing decreased vacuolar degeneration of the hepatocytes (Figures 3C, D, respectively).

The histopathology of the testes of the treatment groups at 15 days post-treatment of the experiment is shown in Figure 4. The testes of the negative group showed typical testicular histology, with sperm affected by interstitial Leydig cells (LC) and spermatogenic cells (SC) lining the seminiferous tubule (Figure 4A). The testes of the control positive group showed spermatogenesis arrest in certain seminiferous tubules accompanied by interstitial edema and spermatogenic cell degenerative alterations (Figure 4B). The testes of the camel milk group showed decreased vacuolar degeneration of the spermatogenic cells with decreased oedema within the interstitial tissues (SC indicates spermatogenic cells and LC indicates Leydig cells) (Figure 4C). The testes of the silymarin group showed a marked decrease in the degenerative changes and mostly revealed typical testicular histology, with sperm affected by interstitial Leydig cells (LC) and spermatogenic cells (SC) lining the seminiferous tubule (Figure 4D).

### Gene expression

**FIGURE 5:**
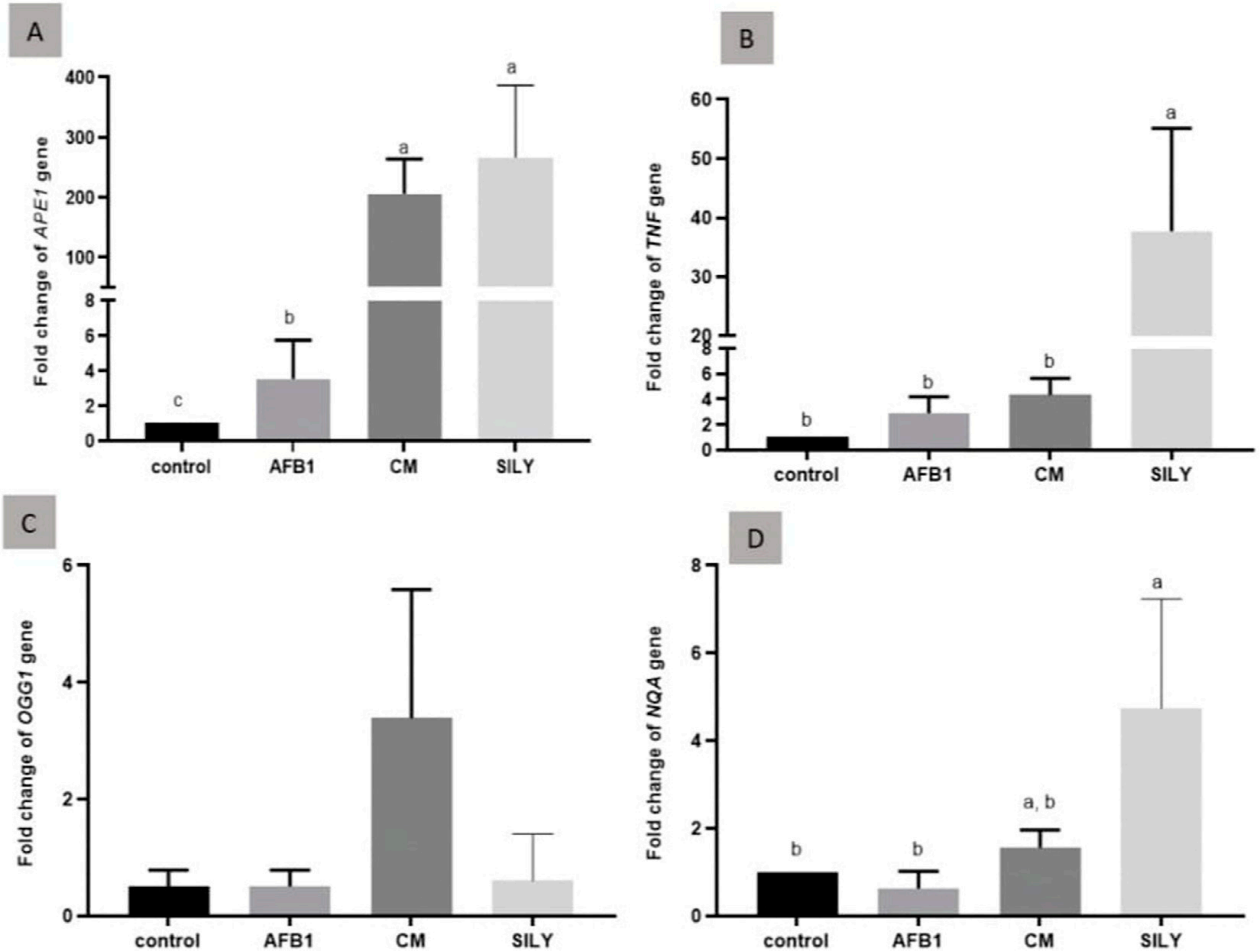
Ameliorative effects of camel milk and silymarin on gene expression in liver tissues. The gene expression (fold change) was calculated relative to the control negative group (calibrator). **(A)** Fold change of APE1 gene, **(B)** Fold change of TNFα gene, **(C)** Fold change of OGG1 gene, and **(D)** Fold change of NQA gene. AFB: Aflatoxin, CM: camel milk, SILY: Silymarin.

As demonstrated in Figure 5, the findings of gene expression were assessed by qRT-PCR, normalized to *β-*actin mRNA of the liver tissue of the experimental groups at 15 days post-treatment. Aflatoxin administration produced a significant (control positive group) downregulation of *TNFα*, *APE1*, and *NQO 1* and a non-significant downregulation of *OGG1*, as opposed to the levels in the control negative group. The treatments (camel milk and silymarin) significantly upregulated the gene expression of *APE1* and *NQO 1* compared to the levels in the positive control group. *OGG1* was insignificantly upregulated, as opposed to the levels in the control negative group. *TNFα* was significantly upregulated in the silymarin group compared to the levels in the control negative and positive groups.

**FIGURE 6:**
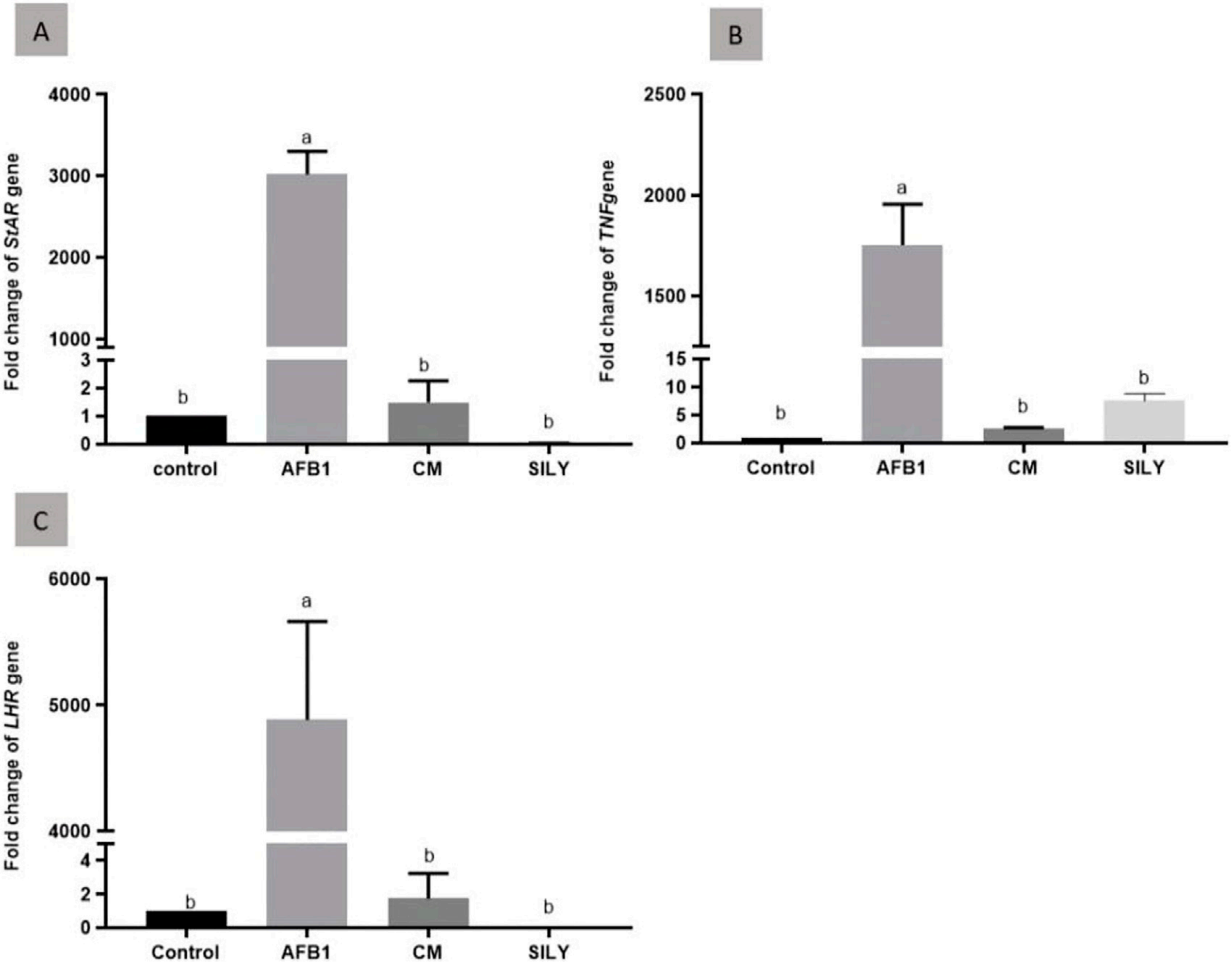
Ameliorative effects of camel milk and silymarin on gene expression in testicular tissues. The gene expression (fold change) was calculated relative to the control negative group (calibrator). **(A)** Fold change of StAR1 gene, **(B)** Fold change of TNFα gene, and **(C)** Fold change of LHR gene. AFB: Aflatoxin, CM: camel milk, SILY: Silymarin.

As demonstrated in Figure 6, the findings of gene expression were assessed by qRT-PCR, normalized to *β-*actin mRNA of the testis tissue of the experimental groups at 15 days post-treatment. As opposed to the control negative group, the injection of aflatoxin resulted in a substantial upregulation of *TNFα* mRNA expression. Camel milk and silymarin administration produced significant downregulation of *LHR1*, *StAR1,* and *TNFα* mRNA expression in comparison with the control negative group.

## Discussion

Previous research has identified the harmful effects of AFB1 on the liver and kidneys, suppression of regular growth, modifications to developmental and reproductive factors, immunotoxicity, and alterations to the gut microbiome of rodents (Li et al., 2022; Sobral et al., 2022). Adverse alterations in renal and hepatic biochemical markers frequently point to liver damage linked to aflatoxin in animals (Aleissa et al., 2020; Hernandez-Valdivia et al., 2021). The present study demonstrated the adverse effects of AF on leukocytic count, which align with Uluişik et al. (2020), Kılıç et al. (2022), and Hassaneen et al. (2023), who reported that compared to the control group, the aflatoxicosis group had a significantly decreased WBC, lymphocyte, and monocyte count. However, these findings could be explained by aflatoxin’s harm to hematopoietic tissue (Pepeljnjak et al., 2003).

There are numerous documented benefits of camel milk, including antiviral, antibacterial, antitumor, antifungal, antioxidant, anti-inflammatory, hypoglycemic, and anti-cancer properties (Behrouz et al., 2022). When given orally for 15 days following treatment, camel milk helped to mitigate the detrimental impact of AFB1 on leukocytic numbers. These findings were in line with Khan (2017), who found that feeding camel milk to mice reduced the leukopenic effect of cyclophosphamide. These results may be attributed to the antioxidant properties of camel milk (Kumarappan et al., 2010). Moreover, Rahman et al. (2005) reported that camel milk contains high levels of vitamins C, A, E, zinc, and magnesium (Abu Zeid et al., 2019). These vitamins and minerals have an antioxidant effect. Several enzymes in the body have been found to be associated with zinc, which can prevent cell damage by strengthening the antioxidant system and its various functions (Ozdemir and Inanc, 2005; Abu Zeid et al., 2019).

Numerous studies have documented the antiviral, anti-fibrotic, anti-proliferative, immunomodulatory, and antioxidant qualities of silymarin. It also has an impact on DNA and RNA synthesis. Moreover, silymarin keeps the hepatocyte membrane intact and prevents harmful compounds or xenobiotics from entering the cell (Karimi et al., 2011). Its supplementation has increased the WBC count in quail (Khazaei et al., 2022). The curative and preventive benefits of silymarin on rat immunological toxicity caused by chlorpyrifos were described by El Elaimy et al. (2013). Since silymarin can stop the generation of free radicals during the metabolism of harmful substances, it possesses hepatoprotective and antioxidant properties (El-Gendy et al., 2024; Zalat et al., 2021).

The liver’s ability to detoxify the body and eliminate contaminants and impurities makes it one of the most important organs in the body (Khan, 2006). The serum biochemical markers, particularly the levels of serum proteins and AST and ALT activities, show how AFB1 affects the liver. Because serum AST and ALT are located in the cytoplasm and are consequently discharged into circulation following cellular damage, they are the most sensitive markers used in the diagnosis of liver damage. The extracellular turnover of these marker enzymes, their subsequent release of proteins, methods of cellular injury, and mechanisms of neoplastic processes are all reflected in the analysis of Jahan et al. (2011). These serum enzyme activity findings are consistent with El-Bahr (2015), Abdel-Wahhab et al. (2016), Abdel-Daim et al. (2021), and Hassaneen et al. (2023), who demonstrated that rats given AFB1 showed significantly higher liver enzymes (ALT and AST) than control. Increases in serum ALT and AST activity are known to be indicative of liver injury.

In this investigation, post-treatment 15 days with camel milk or silymarin to rats was observed to reduce the rise in serum AST and ALT activity brought on by AFB1 treatment. Our findings corroborated those of Althnaian et al. (2013), who observed that camel milk administration to rats reduced the rise in serum AST and ALT activity brought on by CCl4 treatment. This might be due to their antioxidant and anti-inflammatory properties. Legrand et al. (2005) reported that the distinctive anti-inflammatory and antioxidant properties of CM can be explained by its high lactoferrin content. El Mesallamy et al. (2011) demonstrated that in comparison to mice treated with NDEA, silymarin administration significantly decreased blood ALT, AST, and GGT activity. These results may be attributed to the antioxidant effect of silymarin. Silymarin may help the liver’s antioxidant defense by increasing hepatic glutathione (Vargas-Mendoza et al., 2014). In addition, the body weight, histological damage, serum ALT, AST, BUN, SCr, and tissue NO levels were dramatically returned to normal following 7-day post-treatment with silymarin administered 1 h after APAP injection (Bektur et al., 2016). Pradeep et al. (2007) reported that after receiving a single dose of diethyl nitrosamine via injection, the elevated activity of AST and ALT in serum was considerably reduced after 30 days of silymarin treatment.

This study’s results that serum proteins decrease albumin by administration of AFB1 agree with Abdel-Wahhab and Aly (2005), Abdel-Daim et al. (2020a), and Abdel-Daim et al. (2020b), who found that whereas aflatoxin therapy significantly decreased total protein, albumin, and cholesterol, it significantly increased ALT, AST, alkaline phosphatase, total bilirubin, and urea. Total protein levels were significantly lower in rats exposed to AFB1 than in the negative group (El-Bahr, 2015). These could be brought on by AFB1, which disrupts the process of protein biosynthesis by creating adducts with proteins, DNA, and RNA, suppressing the production of RNA and DNA-dependent RNA polymerase activity and inducing degranulation of the endoplasmic reticulum (Mohajeri et al., 2018). In this study, rats given camel milk as a post-treatment for 15 days showed an increase in their serum albumin concentration. These outcomes were accepted by Althnaian et al. (2013), who demonstrated that when compared to rats that got CCl4 alone, rats that received camel milk either alone or in combination showed a significant rise in serum albumin concentration. When camel milk is administered to both humans and animals, there is a potential correlation between the rise in plasma protein thiol activities and the reduction in lipid peroxidation processes, which leads to an increase in albumin concentration (Al-Hashem et al., 2009; Al-Fartosi et al., 2012). Moreover, this might be because of their dietary standards or because of their higher plasma protein thiol activities and lower lipid peroxidation (Sadek et al., 2016; Al-Fartosi et al., 2012). On the other hand, it has been documented that silymarin’s hepatoprotective mechanisms include an increase in hepatic protein synthesis (Popoola et al., 2022; Bijak et al., 2014). Moreover, silymarin’s capacity to lower inflammation (Bahmani et al., 2015) and its antioxidant qualities (Shaker et al., 2010), which actively lower reactive oxygen species and prevent cellular damage, are responsible for the decrease in the concentration of liver enzymes in the blood. It has also been demonstrated that silymarin stimulates RNA polymerase I activity, which in turn promotes protein production in hepatocytes (Vargas-Mendoza et al., 2014).

The aflatoxin group showed a significant decline according to the serum ferritin data. The results of Stoltzfus et al. (2004) affirm these results, stating that a drop in serum ferritin leads to a decrease in serum iron. AFB1 caused a decrease in serum iron, total iron-binding capacity, utilized iron-binding capacity, and transferrin levels (Hassan et al., 2020). However, CEA and AFP are specific markers for liver cancer. Reactive oxygen species are produced more frequently by AFB1, which damages DNA and causes cancer (Albadrani et al., 2024a and b; Huang et al., 2020). The rise in AFP and CEA concentrations is in line with a previous investigation that discovered rats which consumed AFB1 might elevate these levels (Abdel-Wahhab et al., 2006; Abdel-Wahhab et al., 2010; Abdel-Wahhab et al., 2020). According to the results of the current investigation, camel milk and silymarin lower the AFP and CEA levels in rat serum. This agrees with Hassaneen et al. (2023), who reported a substantial drop in AFP and CEA in groups given silymarin and camel milk.

Leydig cells create testosterone, which is necessary for the maintenance of testicular function and the control of spermatogenesis (Payne and Youngblood, 1995; Walker, 2011). The testosterone level analysis results from this study were astonishingly compatible with those of several other researchers. Owumi et al. (2020) revealed that in comparison to control rats, exposure to AFB1 alone caused a considerable drop in the serum levels of testosterone, FSH, and LH. It also caused a concurrent dip in the activities of G6PD, LDH, ACP, and ALP in the testicular tissue. This reduction in testosterone level might be attributed to decreased testes weights reported by Salem et al. (2001) in rabbits. The ameliorative AFB1 effect on testosterone levels is amplified by camel milk and silymarin in this study, which raises the hormone’s serum level. These outcomes concurred with Zakaria et al. (2016) and Gad et al. (2018) that testicular and serum testosterone levels significantly increased in male rats fed camel milk. Rats fed CM produced increased reproductive hormones, had more sperm, and gained weight in their reproductive organs (Zakaria et al., 2016). The high zinc content of camel milk (Zakaria et al., 2016) may be the cause for these outcomes, as zinc is necessary to sustain serum testosterone levels (Shahraki et al., 2015). Zinc deficiency prevents the pituitary gland releasing follicular stimulating and luteinizing hormones, which stimulate the production of testosterone (Egwurugwu et al., 2013). Furthermore, zinc also contributes to the release and operation of the male hormone testosterone through the enzymes that control the arachidonic acid cascade (Boland and Lonergan, 2003). Abedi et al. (2016) discovered that silymarin increased rat testosterone, ascribing this increase to silymarin’s antioxidant properties. Additionally, milk thistle seeds greatly increased the quality and fertility of semen and the concentration of testosterone in rabbit bucks (Attia et al., 2017). Moreover, Faraji et al. (2019) reported that reversing testosterone levels in mice was more possible with silymarin than the cadmium chloride group.

The serum biochemical markers were corroborated by the liver’s histopathology research findings. The findings of the liver pathological changes in the group treated with aflatoxin are consistent with El-Bahr (2015), who found that rats fed AFB1 showed modified lobular architecture and moderate-to-severe degenerative changes in their livers, marked by hepatocytes that seemed to be in vacuoles and edema. In most hepatocytes, there were a significant number of dispersed, isolated necrotic cells (apoptotic cells). Histopathological and ultrastructural analyses of the liver cells of aflatoxicated rats revealed extensive vacuolar degeneration and necrosis signals (Ali et al., 2021; Khatoon et al., 2024).

The liver pathological changes in the silymarin post-treated group parallel the findings of Ahmad et al. (2002), who demonstrated that thioacetamide therapy was significantly reversed in rat livers after receiving post-treatment with Jigreen and silymarin for 21 days. This was indicated by normal central vein, hepatic cells with well-preserved cytoplasm, and a conspicuous nucleus and nucleolus. Rat liver sections treated with silymarin showed a modest improvement in hepatocytes compared to the NDEA-treated group, with fewer cytomegalic, vacuolated hepatocytes and reduced prominence and nuclear vesiculation (El Mesallamy et al., 2011). This improvement might be explained by silymarin’s ability to effectively lower hepatocytes’ intracellular ROS levels, reducing oxidative stress-induced cellular damage. Additionally, it was discovered that silymarin therapy increased hepatic cell proliferation, indicating that improved liver regeneration might aid in replacing the injured liver cells (Wu et al., 2008). On the other hand, the pathological liver changes in the camel milk post-treated group resemble the findings of Ibrahim et al. (2017), who reported that hepatocytes in treated EVOO and camel milk groups were fully protected and that the hepatic architecture was more normal than acetaminophen (APAP)-induced liver toxicity group in mice. Hepatic tissues from rabbits given camel milk exhibited a notable build-up of macrophages and lymphocytes in the parenchyma, granulomatous lesion, and fibrosis (Saleem et al., 2021).

The results of testicular pathological changes at different stages of life are consistent with Laciakova et al. (1995), who demonstrated how long-term exposure to aflatoxins in pigs and rats results in spermatogenic epithelial cell dystrophy and other histological abnormalities in the male reproductive system. Additionally, anomalies in sperm morphology, testis histology, and meiotic chromosomes have been connected to aflatoxin exposure (Sinha and Prasad, 1990). Aboelhassan et al. (2018) revealed that AFB1 treatment resulted in some seminiferous tubules in the testis degenerating compared to the control group. The seminiferous tubule basement membrane buckled, and the quantity of spermatogenic cells decreased, indicating this degeneration. The histological architectural framework of treated rats exhibited significant alterations, as evidenced by necrosis of the tubules in seminiferous and inadequate sperm in the testis, lumen, or epididymis of AFB1 (Owumi et al., 2022). The testicular pathological changes in the camel milk post-treated group parallel the findings of Mohamed et al. (2019), who reported that using CM reversed the toxic effects of fenpropathrin by blocking the action of apoptosis via the caspase 3 and P53 pathways—crucial for the repair of tissue in the testes and other organs. CM group male rats dominated the entirely typical structure of the germinal epithelium in the seminiferous tubules and interstitial tissues, which showed active spermatogenesis and consisted of a regular arrangement of all forms of germ and Sertoli cells (Mohammad et al., 2023). The testicular pathological changes in the silymarin post-treated group resemble the findings of Faraji et al. (2019), who reported that, when comparing the silymarin + cadmium chloride group to the cadmium chloride group in mice, these histological alterations were significantly recovered. Comparing the silymarin-only group to the control group, measurements were made of the diameter of the seminiferous tubule wall, the spermatogonial nucleus, the density of sperm in the lumen, and the regularity of the germ. Additionally, in comparison to the control group, there was a decrease in the interstitial tissue and germinal epithelium vacuolation in the silymarin group. Following the administration of CDDP, silymarin corrected a significant decrease in serum levels of testosterone as well as a significant injury to the testicles and epididymis (El-Hameed et al., 2021).

*TNF-α* is the first and most important inflammatory mediator in the genesis of inflammation (Cruceriu et al., 2020; Jang et al., 2021). *TNF-α* and *NF-B/p65* pathways produce pro-inflammatory cytokines such as *IL-6* and *iNOS*, as well as adhesion molecules that encourage leukocyte migration to the site of inflammation (Arbab et al., 2017; Giridharan and Srinivasan, 2018). *TNF-α* gene expression in liver tissue was downregulated in the present investigation as a result of aflatoxin treatment. However, *TNF-α* gene expression was markedly elevated in camel milk and silymarin after a 15-day post-treatment. These results are consistent with Jiang et al. (2015), who revealed that the expression of *IL-2, IL-4, IL-6, IL-10, IL17, IFN-* γ, and *TNF-* α mRNA was commonly downregulated in the duodenum, jejunum, and ileum of broilers in the AFB1 group. The expression of *TNF-α, IFN-γ,* and *IL-4* was decreased in rats exposed to AFB1, in accordance with Qian et al. (2014).

The aflatoxin-treated group exhibited considerable downregulation of the *OGG1* and *APE1* base excision repair genes, with *OGG1* showing just a little downregulation compared to the control group. These findings support the conclusions of Liu et al. (2018), who showed that AFB1 significantly downregulated the expression of BER genes *OGG1* and *XRCC1*.

In order to protect wholesome cells from oxidative stress and malignancy, *NQO1* is essential. Despite the biological activities of this “cell protector,” *NQO1*’s antioxidant role was paradoxically demonstrated by the evidence demonstrating that genetic variation or disruption of the gene increased the likelihood of chemical-induced toxicity and cancer (Su et al., 2012; Yang et al., 2014; Aboubakr et al., 2021). *NQO1* was notably downregulated in the group treated with aflatoxin in contrast to the other groups. These results are in line with Wang et al. (2022), who demonstrated that the livers of aflatoxicated mice had considerably lower mRNA levels of downstream target genes, including *SOD*, *CAT*, *HO-1*, and *NQO1*. The livers of aflatoxicated mice showed lower expression of the *Nrf2, HO-1, GCLC, NQO1*, and *SOD1* genes (Rajput et al., 2021). In addition, in this study, silymarin post-treated to aflatoxicated rats for 15 days showed significant upregulation of *NQO1* gene expression. Owumi et al. (2020) concluded that exposure to AFB1 alone obviously enhanced *NO, TNF-a*, and *IL-1b* levels in addition to MPO activity and also decreased the anti-inflammatory cytokine, *IL-10*, in the epididymis, testes, and hypothalamus of the treated rats. This result suggests the induction of inflammation. It is commonly known that uncontrolled testis production of cytokines promote inflammatories including *TNF-a* and *IL-1b*, which are detrimental to sperm formation and cause male infertility (Fijak et al., 2018). Nonetheless, TNF-α gene expression was significantly downregulated in the groups that received post-treatment camel milk and silymarin for 15 days. These findings agree with Belhan et al. (2021), who found that the testicular tissue’s expression levels of 8-hydroxy-2-deoxyguanosine (8-OHdG) and tumor necrosis factor alpha (*TNF-α*) greatly rose in the torsion/detorsion groups, and that these levels decreased when silymarin was administered.

*StAR* is usually recognized as the steroidogenesis’ rate-limiting stage and is required for the transport of cholesterols into mitochondria (Hasegawa et al., 2000). The *LHR*-mediated steroidogenic pathway regulates both the expression and activation of *StAR* in Leydig cells (Adedara et al., 2014; Zeng et al., 2018). The downregulation of *StAR1* resulting from the administration of camel milk and silymarin for 15 days following aflatoxin exposure was statistically significant compared to the negative group. Additionally, after 15 days with both treatments, there was a considerable downregulation of *LHR1* compared to the negative group.

## Conclusion

The results of this study indicate that camel milk and silymarin reduce the adverse effects of AFB1 on the rat model’s leukogram, AST and ALT activity, serum proteins, ferritin, alpha-fetoprotein, carcinoembryonic antigen, liver pathology, and gene expression of tumor necrosis factor (TNF-α), antioxidant gene [NAD (P) H quinone oxidoreductase 1 (NQO1)], and base excision repair genes (APE1 and OGG1) in the liver tissue, as well as testosterone and the expression of genes related to luteinizing hormone receptor, steroidogenic acute regulatory protein, and tumor necrosis factor α with normal testicular architecture. Thus, it is possible to conclude that consuming camel milk and silymarin lessened the detrimental effects of AFB1 on the rat model’s leukocytic count, blood biochemistry, liver and testes pathology, and the expression of certain genes.

## Data Availability

The original contributions presented in the study are included in the article/supplementary material; further inquiries can be directed to the corresponding author.
